# Research progress and practical applications of musculoskeletal ultrasound in military training injuries

**DOI:** 10.3389/fmed.2026.1941324

**Published:** 2026-10-01

**Authors:** Shuo Wang, Hanhan Xu, Qianqian Liu, Xiujie Duan, Ying Chen

**Affiliations:** Qingdao Special Servicemen Recuperation Center of PLA Navy, Qingdao, China

**Keywords:** exercise-related injuries, interventional therapy, military training injuries, musculoskeletal ultrasound, novel ultrasound imaging techniques

## Abstract

Musculoskeletal ultrasound (MSKUS) is a non-invasive imaging technique that is gaining increasing attention. The advantages of this approach include the absence of radiation exposure, cost-effectiveness, and the ability to rapidly provide dynamic examination results. Military training constitutes a pivotal mechanism for the enhancement and fortification of combat capabilities, thereby assuming a strategic function in the advancement of military development. In the context of military training, musculoskeletal injuries resulting from physical training and exercise exert a considerable influence on the health of personnel and the efficiency of their training. This has the potential to result in unnecessary non-combat personnel losses and to increase the burden on the military and the nation as a whole. It is important to note that MSKUS has demonstrated significant effectiveness in the diagnosis of common musculoskeletal injuries and holds great value in the monitoring of treatment progress and the assessment of postoperative recovery. In recent years, advances in ultrasound-guided interventions have enabled physicians to achieve a high degree of precision in the diagnosis and treatment of various musculoskeletal conditions, thus rendering it a more accurate and reliable tool. This review summarizes recent advances in the application of MSKUS across multiple disease areas, including muscles, joints, bones, tendons, ligaments, and bursae. It also explores novel ultrasound imaging technologies and the potential for future ultrasound devices on the military battlefield. The objective of this study is to provide an in-depth examination of research progress regarding the application of MSKUS in physical training and sports-related injuries, with a focus on its diagnostic uses, therapeutic significance, and future developments in this field. A thorough analysis of the most recent research findings and clinical application potential will be conducted to elucidate the important role of MSKUS in enhancing diagnostic accuracy and treatment efficacy for military training-related injuries. This, in turn, will improve the quality of medical care and mitigate the impact of training injuries on future military operations.

## 1 Introduction

Musculoskeletal ultrasound (MSKUS) is a valuable diagnostic and interventional tool that is both safe and widely accepted by patients and physicians. It enables dynamic and comparative scanning. This technology facilitates the immediate and direct correlation of images with clinical findings, which is significantly beneficial in the management of musculoskeletal disorders. Technological advancements have enhanced the accessibility of MSKUS in military settings. Consequently, MSKUS is applied to a wide range of musculoskeletal issues, including inflammatory and degenerative diseases, sports injuries, and regional pain syndromes. This article utilizes a comprehensive review of recent research findings to discuss the significant role of MSKUS in military training-related injuries (Figure 1).

**Figure 1:**
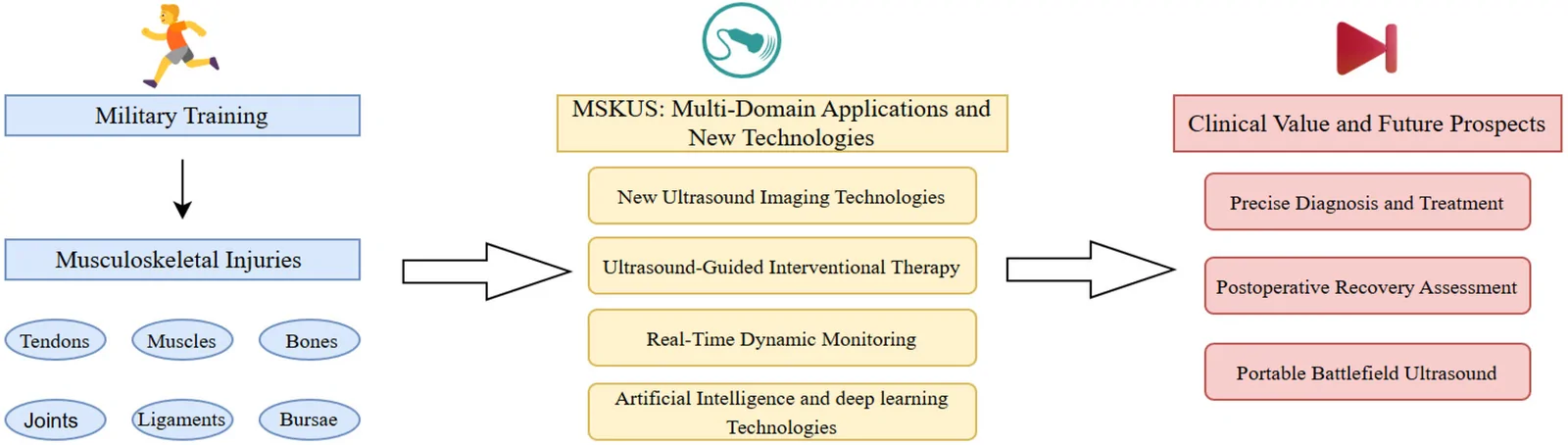
The application of musculoskeletal ultrasound in military training-related injuries.

Search Strategy: This narrative review involved a comprehensive search of literature published between January 1996 and June 2026 in PubMed, Embase, Scopus, and Google Scholar. The search strategy encompassed the following terms and their combinations: A comprehensive search strategy was employed, incorporating terms such as “ultrasound,” “musculoskeletal ultrasound,” “dynamic musculoskeletal ultrasound,” “military training injury,” and “interventional therapy,” along with the logical operators AND and OR. This approach was implemented to maximize the sensitivity and specificity of the results. For instance, the combinations (“Military training injury” OR “Physical training”) and (“ultrasound” OR “Musculoskeletal ultrasound”) were utilized to identify pertinent literature, thereby ensuring the inclusion of relevant, high-quality studies on ultrasound imaging in training-related injuries. The search prioritized literature on the diagnosis, treatment, and application of new technologies using MSKUS in military personnel or individuals undergoing high-intensity physical training. The objective of this strategy was to identify original research, systematic reviews, and meta-analyses to evaluate research progress and the practical utility of musculoskeletal ultrasound in military training injuries.

Inclusion Criteria: The inclusion criteria encompassed both fundamental scientific research and clinical studies. Studies that employed ultrasound to document structural and functional changes, or to evaluate the diagnostic accuracy or clinical utility of ultrasound, were included in the review. The included studies encompassed a wide range of subjects, including musculoskeletal anatomy, histology, imaging, and biomechanics, as well as clinical reports and reviews. Studies were excluded if they lacked imaging data, contained non-peer-reviewed material (such as conference abstracts or commentaries), or were deemed to lack representativeness.

## 2 Overview of musculoskeletal ultrasound technology

### 2.1 Traditional ultrasound technology

Musculoskeletal ultrasound is a noninvasive imaging technique that is becoming increasingly important in the field of medical imaging, particularly in the diagnosis and treatment of musculoskeletal disorders (1). This technique utilizes high-frequency sound waves to generate images of muscles, tendons, ligaments, and joints, thereby enabling the visualization of soft tissue structures. Its noninvasive nature, in conjunction with the absence of ionizing radiation, serves to enhance its appeal within clinical settings. Dynamic musculoskeletal ultrasound (DMUS) provides real-time dynamic views of joints and soft tissues, offering critical insights for the assessment of muscle injuries and exercise-induced injuries (2, 3).

The most commonly used mode in musculoskeletal ultrasound is gray-scale ultrasound, or B-mode (4). Color and power (energy) Doppler modes are superimposed on grayscale images using two colors (typically red and blue), representing blood flow toward and away from the transducer, respectively. Power Doppler has been shown to have higher sensitivity in detecting blood flow in smaller, superficial vessels around joints, thus offering additional advantages in the context of musculoskeletal ultrasound (5). The detection of blood flow via color Doppler or power (energy) Doppler may reveal perfusion characteristics that could be associated with benign or malignant soft tissue masses or with inflammation, such as synovitis within the joint cavity. Doppler imaging facilitates the distinction between joint effusions and hyperechoic or anechoic masses accompanied by neovascularization. This technique also assists the operator in avoiding major blood vessels during intra-articular aspiration and injection (6).

The echogenic characteristics of musculoskeletal structures can be classified into the following types (4): equivalent to soft tissue echogenicity (same brightness), higher than soft tissue echogenicity (brighter), lower than soft tissue echogenicity (darker), or anechoic (appears black). The skin is a uniform hyperechoic layer 1–2 millimeters thick. Subcutaneous fat has lower echogenicity than the skin and exhibits compartmentalized structures. Muscles can be identified by their hyperechoic compartmentalized structures, which are surrounded by the hyperechoic margins of the superficial muscular fascia. Tendons appear as anisotropic hyperechoic fibrous structures. Nerves consist of hypoechoic nerve bundles surrounded by hyperechoic margins. The degree of anisotropy in the epineurium is lower than that in tendons and ligaments. Hyaline cartilage typically appears hypoechoic or anechoic, whereas fibrocartilage appears hyperechoic. The synovial membrane or synovial hyperplasia typically appears hypoechoic. Fluid appears anechoic, but may appear hypoechoic if there are floating debris in the effusion. Finally, the cortical bone appears hyperechoic.

Ultrasound offers the crucial advantage of enabling dynamic assessment of musculoskeletal structures during movement. Advancements in ultrasound-guided interventions for the musculoskeletal system have enabled physicians to achieve a high degree of precision in the diagnosis and treatment of various musculoskeletal conditions, including tumors, inflammation, degenerative arthritis, tendinopathies, and bursal pathologies (7). In recent years, ultrasound-guided interventional therapy (8)—with its common indications and injection sites (Table 1)—has gained widespread clinical recognition for its ability to provide real-time guidance for minimally invasive treatments through precise localization and to dynamically monitor rehabilitation progress. This non-surgical injection therapy technique involves the administration of small quantities of solution via multiple treatments. The solution is injected into sites of pain and degenerative tendons (ligaments), joints, ligaments, and adjacent joint spaces. This process stimulates the proliferation of normal cells and tissues, contributing to the healing process. Moreover, research has demonstrated that ultrasound-guided injections tend to be more precise than those guided by surface anatomical landmarks (16). In the upper extremity, ultrasound-guided injections have been demonstrated to be more efficacious than injections guided by surface anatomical landmarks at the supracondylar region, subacromial space, biceps tendon sheath, and wrist joint. In the lower extremity, ultrasound-guided injections at the knee, ankle, and foot have been shown to be more accurate than those guided by surface anatomical landmarks.

**Table 1:** Indications and injection sites for ultrasound-guided interventional procedures.

Body part	Common diseases	Common injection sites
Shoulder	Rotator cuff tendinopathy, Shoulder impingement syndrome (9)	Attachment sites of the supraspinatus, infraspinatus, and teres minor muscles; attachment site on the coracoid process; medial glenohumeral ligament
Elbow	Lateral epicondylitis (10)	Circular ligament, lateral collateral ligament, radial collateral ligament, radial head, joint capsule, medial and lateral condyles
Back/Hip/Buttocks	Lower back pain (11), Piriformis syndrome (12)	Intertransverse ligaments of the lumbar spine, sacroiliac ligaments/joints, gluteal muscle insertions, and insertions of the external rotator and abductor muscles
Knee	Knee osteoarthritis (13, 14)	The adductor muscles of the thigh, the insertion of the medial head of the rectus femoris, the origin and insertion of the collateral ligament, and the knee joint capsule
Ankle/Foot	Achilles tendinopathy, Plantar fasciitis (15)	Achilles tendon, calcaneonavicular ligament, tarsometatarsal ligament, tibio-talar ligament, tibio-calcaneal ligament

With advances in technology, MSKUS has become an important tool in several medical fields, including sports medicine (17), rheumatology (18, 19), and orthopedics (20, 21). Compared to other imaging modalities such as magnetic resonance imaging (MRI) and computed tomography (CT), ultrasound offers several advantages as a diagnostic tool for musculoskeletal injuries. These advantages include lower cost, shorter scan times, reduced metal artifacts, the portability of ultrasound devices, the absence of radiation compared to CT, and the ability to compare findings with the contralateral side, which is typically normal. Due to its safety and non-invasive nature, ultrasound is particularly well-suited for examinations involving infants, young children, and pregnant women (22, 23); it can also be used for patients with contraindications to MRI.

### 2.2 New ultrasound imaging technologies: technological innovation and clinical practice

#### 2.2.1 Contrast-enhanced ultrasound and microvascular imaging

Doppler ultrasound can be used to evaluate tissue vasculature and detect inflammatory conditions. However, Doppler has limited sensitivity when assessing slow blood flow. Contrast-enhanced ultrasound (CEUS) enhances the ability of ultrasound to assess tissue perfusion (24). During CEUS, a contrast agent consisting of microbubbles is administered intravenously, and the relevant body region is imaged using ultrasound. After injection, perfusion can be monitored by acquiring a sequence of ultrasound images to assess blood flow patterns (25). CEUS assessment of perfusion can evaluate the presentation of inflammatory arthritis in diagnosis, disease progression assessment, and evaluation of treatment response. CEUS is more sensitive than gray-scale and power Doppler ultrasound in the postoperative monitoring of patients with rheumatoid arthritis (26). CEUS is more effective than gray-scale ultrasound and power Doppler in distinguishing between active synovitis and inactive synovial thickening (27). Previous studies have used CEUS to quantify perfusion patterns in soft tissue masses and to assess vascular conditions in repaired rotator cuff tendons (28, 29). To overcome the limited sensitivity of Doppler imaging and the need for contrast-enhanced ultrasound, superb microvascular imaging (SMI) was developed. This technique has the capacity to visualize microvascular flow in soft tissues and has the potential to be utilized in the study of muscle tissue pathology and tissue healing. Doria et al. demonstrated that contrast-enhanced power Doppler ultrasound significantly improves the identification of proximal femoral vascular conditions and can be used to visualize the revascularization process (30). However, given its inability to differentiate between the various stages of revascularization with the same degree of effectiveness as the gold-standard radionuclide scan, its clinical application remains constrained.

#### 2.2.2 Strain elastography and shear wave elastography

Strain elastography (SE) and shear wave elastography (SWE) have been utilized to evaluate a variety of tendons and muscles, including the Achilles tendon, patellar tendon, quadriceps tendon, medial collateral ligament tendon, and rotator cuff tendons and muscles (31). Their efficacy in the early diagnosis, outcome tracking, and monitoring of tendon injury treatment has been well-documented (32). The majority of literature on elastography for musculoskeletal injuries focuses on tendon evaluation, particularly the Achilles tendon. Research has demonstrated a strong correlation between strain elastography and histological evaluations of tendon lesions, as well as with clinical indicators of tendon pain and dysfunction (33). The combination of gray-scale ultrasound analysis and acoustic elastography has been shown to enhance diagnostic accuracy for carpal tunnel syndrome (34). A notable constraint of strain elastography is that the measurements are not inherently quantitative and thus, are inherently subjective.

Shear-wave elastography is a method of measuring the speed of automatic pulses as they travel through tissue (35). The velocity of shear waves is directly proportional to the stiffness of the tissue. Shear wave velocity (V), measured in meters per second (m/s), is related to elasticity via the Young's modulus equation, where elasticity (E)=3*ρ*V2. A substantial body of research has validated the reproducibility of SWE in the evaluation of tendons, ligaments, and muscles (36–38). SWE can quantitatively assess plantar fascia stiffness, which is reduced in patients with plantar fasciitis (39). The primary advantage of SWE lies in its capacity to discern tissue alterations that elude detection in conventional gray-scale ultrasound images. However, the accuracy of SWE measurements is compromised by the influence of sensor pressure and alterations in patient posture, resulting in variability in the output (40).

#### 2.2.3 Overview of existing battlefield-adapted ultrasound devices

In recent years, progress has been made in the development of portable ultrasound systems tailored for military operational environments (41). During Operation Iraqi Freedom (2003–2004), the forward deployment of portable ultrasound demonstrated its potential clinical value through multiple case reports (42): the U.S. Army Combat Support Hospital (CSH) in Mosul performed more than 400 on-site examinations within six months; in British operational areas, handheld focused assessment with trauma ultrasound (FAST)/E-FAST was incorporated into the initial assessment in certain cases and was reported to influence preoperative decision-making, on-site diagnosis, and timely management. Taken together, these early operational experiences indicate that point-of-care ultrasound (POCUS) is feasible and clinically meaningful in specific contexts (43, 44).

The battlefield is a harsh environment for ultrasound machines (45). During the wars in Iraq and Afghanistan, significant fluctuations in ambient temperature led to battery degradation. The environment was also sandy and dusty, causing overheating. Space in medical facilities was limited, and medical personnel might carry small, portable ultrasound devices in their backpacks to the site of injury (46). Ultrasound systems must be handheld, battery-powered, durable, and capable of withstanding extreme temperatures and altitudes; they may also require additional equipment to help prevent battery degradation and device damage. POCUS is portable and lightweight, making it suitable for screening a variety of conditions commonly encountered in harsh environments, disaster situations, and resource-limited settings. Common POCUS applications in harsh environments include eFAST scans, musculoskeletal and soft tissue applications, HAPE assessment, high-altitude cerebral edema, pneumonia, volume status assessment, and various procedure-guidance applications. Various POCUS applications used for surgical guidance in harsh environments include peripheral vascular access, nerve blocks for pain control, foreign body identification and removal, and abscess identification and drainage. POCUS is a reliable tool that aids in patient triage, resource allocation decisions, disease screening, and management of conditions commonly encountered in challenging environments.

## 3 Common injuries related to physical training and sports

Military physical training and sports are fundamental means for developing and enhancing military strength. During military training, the wide variety and high intensity of training exercises place high demands on trainees' physical fitness, and injuries may occur during high-intensity training. Injuries sustained during military training have a serious impact on soldiers' health as well as on the efficiency and effectiveness of training, leading to unnecessary non-combat personnel losses and increasing the burden on the military and the nation (47). Research reports indicate that the overall incidence rate of military training-related injuries in China ranges from 7.43% to 60.7%, with a concentration of 10% to 20%, and that incidence rates vary across different military branches, disciplines, and time periods (48). Among them, the incidence of military physical training and sports-related injuries among recruits is as high as 50%–60%. Obstacle courses, physical training, and tactical training are the disciplines most prone to injuries, with the highest number of injuries occurring during the fatigue period, 6 to 8 weeks after training. Furthermore, harsh environments and dangerous terrain expose individuals to the risk of injury; in such cases, timely and accurate diagnosis and treatment plans are crucial for soldiers' recovery. According to *the Diagnostic Standards and Principles for the Prevention and Treatment of Military Training Injuries* and the *Notice on the Monitoring and Reporting of Military Training Injuries*, military training injuries can be classified into five categories (see Table 2). Among these, soft tissue injuries, as well as bone and joint injuries, are the most common (49).

**Table 2:** Categories of military training injuries.

Category	Contents	Examples of diseases
Soft tissue injuries	Injuries to muscles, ligaments, tendons, fascia, synovial membranes, etc.	Achilles tendinitis, anterior cruciate ligament injury
Bone and joint injuries	Acute fractures, stress fractures, joint dislocations, etc.	Rotator cuff tears, foot and ankle fractures
Organ injuries	Functional and structural damage to the affected organs	Intestinal torsion, concussion
Injuries in special environments	Heatstroke, frostbite, acute mountain sickness, etc.	Exertional heatstroke, high-altitude cerebral edema
Others	Chemical poisoning, burns, bites, and stings, etc	Snake bites, red fire ant stings

More importantly, the vast majority of injuries related to military training occur in the musculoskeletal system (50, 51), and once military physical training and sports-related injuries occur, they can seriously compromise soldiers' health (52). The causes of musculoskeletal injuries are wide-ranging; according to surveys, the primary causes include physical training (PT), sports, field training, chronic diseases, garrison activities, and motor vehicle accidents (53). PT is the primary cause of musculoskeletal injuries. Among sports-related injuries, the majority are caused by running, followed by weight training. In basic training, injuries most commonly occur in the lower extremities, followed by the trunk and upper extremities. The top three specific injury sites, in order, are the foot/ankle joint, the knee joint, and the lower leg. The most common site of training injuries in the trunk is the lower back, primarily manifesting as lumbar muscle strain and lumbar disc herniation. The most common site of training injuries in the upper extremities is the shoulder, primarily manifesting as rotator cuff injuries, among others (54). Military training, characterized by its high-intensity and prolonged nature, renders soldiers susceptible to both acute injuries and chronic overuse injuries. At present, the most prevalent reason for military personnel to seek medical care is musculoskeletal injuries (55). Musculoskeletal injuries resulting from military training are direct factors that limit the mobility of officers and enlisted personnel, cause non-combat casualties, and reduce their combat effectiveness. Rapid and accurate diagnosis of these injuries is a critical step in facilitating subsequent treatment and rehabilitation.

## 4 The application of musculoskeletal ultrasound in common military training injuries

Accurate assessment and diagnosis of injuries form the foundation of triage and treatment (56, 57). MSKUS facilitates the visualization of movement and function in various structures, including bones, muscles, and joints, during movement. Conventional imaging techniques, including x-rays and CT scans, yield static images of fractures; however, they lack the capacity to assess the functional state of injured muscles. Conversely, MSKUS facilitates the observation of muscle behavior during movement, thereby enabling real-time assessment of the extent of skeletal and muscular injuries and monitoring of the recovery process (58). The utilization of ultrasound is a pervasive phenomenon, owing to its portability, safety, real-time imaging capabilities, and expeditious data collection. Given the pivotal role of musculoskeletal ultrasound in the diagnosis and treatment of musculoskeletal disorders, its application to military training injuries has emerged as a new area of research (59). However, it is imperative to acknowledge that MSKUS does not constitute a universal solution. In clinical decision-making regarding military training injuries, MSKUS should be appropriately selected for rapid dynamic assessment based on the specific injury site and pathological type. When bony structures, deep intra-articular lesions, or precise preoperative anatomical localization are involved, MRI, CT, or x-ray examinations are still required to obtain more comprehensive anatomical and pathological information (Table 3).

**Table 3:** Applications of musculoskeletal ultrasound in major anatomical structures.

Anatomical structure	Normal ultrasound findings	Common pathological changes associated with military training injuries	Diagnostic value of MSKUS	Limitations
Tendons	A fibrous, hyperechoic structure with internally parallel, fine, thread-like echoes; a small amount of synovial fluid is visible around it	Tendinitis, tenosynovitis, tendon tears, Achilles tendinopathy, rotator cuff injuries	Superficial tendons are clearly visualized, and dynamic scanning can assess tendon gliding function; color Doppler can detect new blood vessels	Bone acoustic shadows obscure structures behind bony attachment sites; anisotropy causes angle-dependent measurement errors in the sound beam
Muscles	Fibrillar hyperechoic streaks against a hypoechoic background; the feather-like muscles show a characteristic oblique arrangement	Muscle contusions (hematomas, torn muscle fibers), muscle strains	Real-time dynamic assessment of muscle contraction function allows for monitoring of hematoma resorption and muscle fiber regeneration	Imaging quality of deep muscles (such as the gluteus minimus and psoas major) is affected by body type; the limited field of view makes it difficult to fully visualize extensive muscle injuries.
Bones	The cortical bone appears as a continuous, hyperechoic line with an acoustic shadow behind it.	Stress fractures, bone contusions, periosteitis	Shows early periosteal thickening and subperiosteal hematoma; color Doppler can detect increased blood flow at the fracture site and monitor callus formation.	Ultrasound cannot penetrate the cortical bone; it has low sensitivity for detecting early-stage stress fractures.
Ligaments	A thin-layer, hyperechoic, band-like structure with a well-defined course that appears linear under tension	Ligament sprains, partial/complete tears, ligament laxity, and lateral ankle ligament injuries	Shows ligament continuity, thickness, and echogenic changes; dynamic stress testing can assess ligament tension and joint stability	Assessment of deep ligaments is limited by penetration depth
Joints	A small amount of anechoic synovial fluid is visible in the joint space.	Joint effusion, osteoarthritis	Detect joint effusion and guide aspiration; perform dynamic scanning to assess joint range of motion	Inability to adequately assess deep intra-articular structures
Bursae	A thin-walled, anechoic structure	Bursitis, Synovitis	Energy Doppler can assess the degree of synovial inflammation	The bursa structure located behind the bony articular surface, obscured by an acoustic shadow

### 4.1 Tendons

The term “tendinopathy” is commonly used to describe clinical manifestations of localized tendon pain, accompanied by pain on loading, tenderness on palpation, and/or impaired function ^(^60). It is a very common musculoskeletal condition that frequently affects the Achilles tendon, patellar tendon, and rotator cuff tendons (61). MSKUS technology offers diagnostic advantages for these conditions. Tendon abnormalities observed via ultrasound in asymptomatic tendons can predict future tendinopathy. Identifying high-risk individuals using screening tools such as ultrasound may help facilitate the implementation of prevention programs (62). STEINBERG et al. found that evaluating the structural integrity of soldiers' Achilles and patellar tendons using ultrasound demonstrated that military training, which increases physical stress on musculoskeletal structures, elevates the risk of orthopedic injuries; conversely, proprioceptive prevention programs designed to reduce stress and improve function may be beneficial for tendon health (63).

In the diagnosis of tendon ruptures, musculoskeletal ultrasound (MSKUS) provides real-time morphological and functional information on the injured tendon by observing changes in tendon movement, thereby aiding in the precise localization and assessment of the severity of the rupture and the healing process. Recent studies have shown that musculoskeletal ultrasound is effective in assessing muscle strength deficits following Achilles tendon ruptures and in guiding rehabilitation therapy. In addition, common sports injuries such as muscle strains and tenosynovitis can also be monitored in real time, helping physicians guide treatment (64). In cases of Achilles tendinopathy, MSKUS can reveal the morphology and changes in the tendon during movement. By comparing dynamic images of the injured and healthy tendons, physicians can clearly determine the presence and severity of the lesion. Furthermore, MSKUS supports the quantitative assessment of the mechanical properties of the damaged tendon, such as modulus of elasticity and strain rate, providing a foundation for customized treatment plans.

### 4.2 Muscles and bones

Muscle injuries, which are also common in military training and sports, are caused by contusions, strains, or tears; more than 90% of sports-related injuries are contusions or strains (65). Musculoskeletal ultrasound plays an important role in the diagnosis and assessment of muscle injuries. MSKUS allows for real-time observation of dynamic changes in the quadriceps femoris, providing clinicians with more intuitive information about the condition (66). In addition, MSKUS can assess muscle contraction function and tendon gliding, aiding in the diagnosis and evaluation of the severity of muscle injuries (67)

Among new recruits, the most common type of military training-related injury is a stress fracture (68). Studies have shown that ultrasound is highly effective in assessing long bone fractures, with good diagnostic accuracy: 93% sensitivity and 92% specificity for the upper extremities; and 82% sensitivity and 93% specificity for the lower extremities (69). As with most single-region ultrasound examinations, the assessment can be completed within 5 min. Even more notably, studies have found that MSKUS offers unique advantages in fracture localization and quantitative assessment (70). By observing the movement and deformation of musculoskeletal tissues in real time, it accurately assesses the stability and healing status of fracture sites. Following surgical fixation of fractures, MSKUS assists physicians in observing the precise position of fixation screws or plates, identifying any issues—including loosening or displacement—and guiding subsequent treatment measures (71). Furthermore, MSKUS can assess the extent of ligament and muscle injuries, which is critical for the recovery of joint function following fractures. Minimally invasive techniques are becoming increasingly common in orthopedic surgery, such as arthroscopy and endoscopic soft tissue repair; given the extremely high demands of these procedures, highly precise visual guidance is essential. DMUS provides real-time 3D imaging, helping surgeons precisely locate and quantitatively assess key structures during surgery, such as fracture fragments, the patellar ligament, and tendons. During arthroscopic chondroplasty, high-definition ultrasound technology helps surgeons observe changes in the cartilage surface and the effects of repair, enabling them to adjust the surgical plan in a timely manner and thereby improve the success rat (72). Ultrasound can serve as a first-line screening tool for fractures, particularly in emergency departments, on the battlefield, in disaster areas, or in other situations where x-ray equipment is unavailable, for rapid preliminary assessment rather than definitive diagnosis. Currently, plain x-rays remain the most commonly used first-line diagnostic method for fractures.

### 4.3 Joints and ligaments

In the context of military training, shoulder injuries occur at a high rate among individuals engaged in physical training and sports, significantly impacting the training effectiveness of officers and soldiers as well as the combat readiness of military units (73). There are many causes of shoulder injuries, among which rotator cuff injuries are relatively common. The common causes of rotator cuff injuries in routine military training primarily include acute rotator cuff tears (RCT) and chronic overuse injuries. Acute tears are commonly caused by sudden, forceful lifting of heavy objects, using the shoulder to brace during a fall, or traction from external force. Chronic overuse injuries typically result from previous falls, bracing the upper limbs against the ground, or straining to lift heavy objects. In addition, during training for explosive upper-arm strength exercises—such as throwing events, horizontal bar exercises, and rings—excessive force applied to the upper limbs without adequate warm-ups or proper technique can easily lead to rotator cuff injuries. This poses a significant challenge for traditional diagnostic methods such as x-rays and MRI, as these techniques may fail to effectively reveal the dynamic structure and function of the rotator cuff. In contrast, musculoskeletal ultrasound is a recognized method for RCT diagnosis (74), MSKUS can visualize injuries to structures such as the shoulder joint capsule, glenoid labrum, and long head of the biceps tendon, as well as the stability of the humerohumeral joint. By providing real-time visualization of dynamic changes in the rotator cuff—such as bursitis, tendinitis, and tears—it offers clinicians more intuitive information about the condition, thereby guiding the development of clinical treatment plans (75).

The knee joint is one of the most commonly affected joints in musculoskeletal injuries. Studies have shown that MSKUS can effectively monitor the progression of pathological conditions in the knee joint, such as fluid accumulation, synovitis, meniscal tears, and ligament injuries (76, 77). In addition, it allows for dynamic observation of changes in articular cartilage, the joint capsule, and ligaments during joint movement (78). A survey found that among military training injuries, knee injuries caused by ligament injuries accounted for 28.6% of cases, with anterior cruciate ligament (ACL) injuries being the most common. According to literature statistics, the incidence rate of anterior cruciate ligament injuries among male military personnel is 3.79‰; most require inpatient surgical treatment following injury, which significantly impacts routine military training (79). In the diagnosis of ACL injuries, MSKUS can clearly display the ligament's morphology, position, and continuity, as well as adhesions between the ligament and surrounding soft tissues, thereby aiding in localization, qualitative assessment, and staging (80). Currently, in the field of ACL injuries and reconstruction, magnetic resonance imaging (MRI) is the tool of choice for confirming ACL rupture and assessing potential associated injuries (81). However, MSKUS also holds value during the course of treatment. For knee disorders, it helps monitor the resorption of synovial fluid, the repair of articular cartilage, and the healing of the meniscus, thereby guiding the formulation of clinical treatment plans. For example, in the treatment of knee synovitis, MSKUS can monitor the resorption of synovial fluid in real time, evaluate treatment efficacy, and guide clinical medication and physical therapy (8).

### 4.4 Bursae

Bursae are sacs located outside joints, which may communicate with the joint; their function is to reduce friction between tendons and bones or skin and to facilitate the movement of muscle-tendon structures (82, 83). Inflammation of these structures, typically caused by increased friction or trauma, can be a source of pain and functional impairment. In chronic bursitis, the synovial wall of the bursa may thicken due to proliferative tissue, and may be accompanied by calcifications and internal hyperechoic debris. Distinguishing between infectious, metabolic, or inflammatory bursitis in these cases can be challenging; however, ultrasound-guided aspiration for fluid analysis can help resolve this clinical dilemma. With advances in this field, combining MSKUS findings with metabolic and inflammatory biomarkers may support the pathophysiological classification of various diseases, paving the way for more personalized interventions (84). Furthermore, ultrasound-guided corticosteroid injections are an important method for treating shoulder pain; one of the most common injection sites is the subdeltoid bursa, which is used to treat acromioclavicular impingement syndrome. Ultrasound-guided bursitis injections are more accurate than palpation-guided injections, and some studies suggest that ultrasound-guided injections result in significantly better clinical improvement (85, 86).

### 4.5 Other

In recent years, the continuous upgrading of ultrasound equipment and technological advancements have led to the widespread application of MSKUS in the diagnosis and treatment of soft tissue diseases. MSKUS has demonstrated particularly outstanding performance in the detection of soft tissue tumors (87). Lipomas are the most common benign soft tissue tumors, frequently occurring in the limbs and trunk. MSKUS can clearly depict a tumor's morphology, borders, internal echogenicity, and adhesion to surrounding tissues. This aids in localization, characterization, and staging. Hemangiomas are benign tumors caused by the abnormal proliferation of vascular endothelial cells. MSKUS can display blood flow signals within hemangiomas and delineate the borders between the tumor and the surrounding tissues. This facilitates diagnosis and differential diagnosis (88, 89). Additionally, MSKUS is used to diagnose malignant tumors. Sarcomas originate in muscle tissue and commonly occur in the extremities and trunk. MSKUS can assess their morphology, borders, and internal echo characteristics, as well as the tumor's attachment to surrounding tissues. This facilitates the localization, characterization, and staging of sarcomas (90).

MSKUS is primarily used to assess soft tissue tumors preoperatively and postoperatively and to monitor treatment efficacy. By observing real-time changes in tumor morphology, size, location, and attachment to surrounding tissues, MSKUS provides critical information for developing clinical treatment plans. For lipomas, MSKUS helps monitor changes in tumor volume and evaluate treatment efficacy, guiding decisions regarding medication and physical therapy. Furthermore, in the treatment of hemangiomas, MSKUS tracks changes in tumor blood flow signals, assesses treatment efficacy, and supports surgical and medical decision-making.

## 5 Discussion

As modern warfare increasingly emphasizes information superiority, multi-domain coordination, and asymmetric hybrid operations, the battlefield has become an unpredictable, operationally complex, and high-intensity environment (91). Military training plays a strategic role in enhancing and strengthening combat capabilities and driving military development. Consequently, medical support systems must evolve to include higher levels of intelligence, a modular design, and forward deployment. Musculoskeletal ultrasound, which provides radiation-free imaging, portability, and real-time diagnostic capabilities, plays an increasingly important role in military physical training and in diagnosing and treating sports-related injuries.

Traditional medical imaging methods, such as x-rays and CT scans, have limitations regarding their size, difficulty of deployment, and radiation risks. These limitations may restrict their use in mobile and rapid-response scenarios. Ultrasound imaging, on the other hand, offers a non-ionizing, real-time, and portable alternative. Ultrasound imaging is an indispensable tool for diagnosing musculoskeletal conditions resulting from military training injuries, and its importance in clinical practice cannot be overstated. MSKUS aids in accurately diagnosing various conditions, such as tendon tears, bursitis, and joint effusions, and it guides interventional procedures, such as injections and aspiration (7, 92). Furthermore, several studies suggest that musculoskeletal ultrasound will become widespread in the field of rehabilitation (93). Assessing muscle strength and function recovery during rehabilitation is crucial, and MSKUS enables real-time observation of muscle contraction and relaxation. This helps clinicians evaluate patients' recovery of muscle strength and coordination. MSKUS is particularly useful during rehabilitation following anterior cruciate ligament (ACL) reconstruction of the knee. It assists physical therapists in quantitatively assessing the contraction of the patient's thigh muscles. This allows for timely adjustments to the rehabilitation plan, improving treatment outcomes.

Recent research shows that the integration of artificial intelligence into MSKUS is paving the way for enhanced diagnostic capabilities, enabling healthcare professionals to interpret ultrasound images more accurately and efficiently (94, 95). For example, the use of musculoskeletal ultrasound to examine the muscles and bones of patients with supraspinohumeral arthritis, combined with artificial intelligence algorithms, represents a new tool for diagnosing and staging this condition (96). A variety of AI-based technologies can improve the accuracy of assessments. These include: automated image acquisition, image interpretation, anatomical landmark detection, measurement and quantification, probe positioning guidance, elastography interpretation, and fusion with other imaging modalities. Future developments will focus on advancing AI-assisted diagnosis, integrating physiological monitoring and communication modules, and enhancing adaptability to extreme environments, thereby facilitating the transition of military medicine from a tool-based practice to a systems-oriented approach. Future diagnosis and treatment will evolve toward individualization, precision, intelligence, and multidisciplinary collaboration, requiring strengthened military-specific clinical research and the development of dedicated databases. Advances in specialized military ultrasound systems and their systematic deployment may become a key strategic direction for enhancing future combat medical readiness.

Although MSKUS plays an important role in sports medicine diagnostics, it still has certain limitations. As is the case with the majority of imaging modalities, a learning curve is associated with the technical skills and the ability to use the equipment accurately (97). In contrast to CT or MRI, MSKUS is a manual examination that is highly dependent on the operator's experience. The absence of standardized, formal training can potentially contribute to an increased frequency of misdiagnosis or overlooked diagnoses. Secondly, the principles of physical imaging impose dual limitations on both the field of view and depth. The signal-to-noise ratio of ultrasound imaging for deep structures—such as deep hip structures or paravertebral structures in obese subjects—is significantly diminished, and body conformation directly limits its penetration. Concurrently, the acoustic shadow cast by the smooth, hyperechoic surface of bones generates “blind spots” in the tissues posterior to them. Consequently, MSKUS is not a substitute for MRI or CT in evaluating specific intra-articular structures that cannot be adequately assessed, such as those involving cortical continuity, deep intra-articular loose bodies, or the labrum. In the final analysis, the physical size limitations of the equipment and probes make it difficult to balance high resolution with wide-area scanning when evaluating small joints or extensive muscle strains. In circumstances involving bony structures, deep intra-articular lesions, or cases necessitating precise preoperative anatomical localization, MRI, CT, or x-ray examinations remain essential to acquire comprehensive anatomical and pathological information. In the future, with the development of new technologies such as elastic imaging and microvascular imaging, as well as advances in equipment miniaturization, MSKUS is expected to see further expansion in the diagnosis of common musculoskeletal injuries, as well as in monitoring treatment progress and evaluating postoperative recovery.
